# Temperature Effects on Effluent Microgel Formation

**DOI:** 10.3390/polym14224870

**Published:** 2022-11-11

**Authors:** Hsiao-Ming Chang, Carlos I. Vazquez, Ruei-Feng Shiu, Wei-Chun Chin

**Affiliations:** 1 Department of Bioengineering, School of Engineering, University of California at Merced, Merced, CA 95343, USA; 2 Quantitative and Systems Biology, School of Engineering, University of California at Merced, Merced, CA 95343, USA; 3 Institute of Marine Environment and Ecology, National Taiwan Ocean University, Keelung 202301, Taiwan; 4 Center of Excellence for the Oceans, National Taiwan Ocean University, Keelung 202301, Taiwan

**Keywords:** WWTP effluent, dissolved effluent organic matter, microgel, aggregation, temperature

## Abstract

Wastewater treatment plant effluent is considered an important hotspot of dissolved organic matter. The behavior and transformation of dissolved effluent organic matter (dEfOM) regulate particle sedimentation, pollutant fate, microbial attachment, and biofilm formation. However, studies have so far focused on the transformation of marine and riverine organic matter, and the current knowledge of dEfOM behavior is still limited. Fluctuations in water conditions, especially temperature, may directly alter the size, assembly speed, and structure of microgels, thereby potentially disturbing fate and the transportation of organic matter. In this study, we firstly investigated the effects of temperature on the behavior and capacity of dEfOM assembly into microgels and the possible mechanism. The microgel size and granularity of dEfOM were monitored by flow cytometry. Our results suggest that, with regard to microgels, a higher temperature leads to a higher assembly capacity but also a decrease in the size distribution. By contrast, assembly at 4 °C reduces the relative assembly capacity but increases the microgel size and granularity. The size distribution of the formed microgels at the various temperatures was ordered as follows: 4 °C > 20 °C > 35 °C. The size reduction in dEfOM assembly may be closely tied to the enhancement of hydrophobic interactions. The reduction in microgel granularity in warm conditions (35 °C) in terms of the effluent water may be caused by thermally induced condensation. Overall, the findings demonstrate the effects of temperature on dEfOM assembly and can facilitate further relevant studies on aquatic organic particle formation during current global warming scenarios.

## 1. Introduction

Dissolved organic matter (DOM) exists in aquatic systems as one of the most complex chemical mixtures. DOM generally consists of substances that can pass through a 0.22 or 0.45 μm filter (in this study, a 0.45 μm filter were used) [1]. It is composed of multiple carbon-based compounds, including humic acids, fulvic substances, soluble microbial products, biological secretions, and organic pollutants, that derive from bacterial activity, photosynthetic production, terrestrial input, and industrial effluents [2,3,4]. The role of DOM materials in the environmental can be extensive, as they actively participate in numerous biogeochemical processes, such as element exchange, microbial loops, pollutant mobility, and carbon sequestration [5,6,7]. Previous studies have indicated that DOM contains polysaccharides, proteins, lipids, nucleic acids, and various functional group types (carboxylates, sulfates, and phosphates) that can possibly provide stable binding and adsorbing sites for organic toxins and metal ions [6,7]. DOM can therefore act as a vital regulator of pollutant toxicity and, in turn, as a modulator of the bioavailability and bioaccumulation of pollutants in organisms in aquatic systems [8,9,10]. For example, the dissolved effluent organic matter (dEfOM) input from wastewater treatment plants (WWTPs) can significantly enhance the DOM contributions of a river to sea systems [6,7]. Similarly, human alterations of/impacts on environmental conditions lead to distinct DOM pools in freshwater, and the levels of anthropogenic microbial humic-like compounds increase significantly in freshwater systems [2,3,11]. In addition, the presence and characteristics of dEfOM can dominate the discharge quality and ultimate fates of DOM as well as its interactions with surrounding materials. The characteristics, biotoxicity, and other interactions of dEfOM have been widely studied in the literature [12,13,14]. By contrast, a comprehensive understanding of dEfOM transformation, particularly regarding the size dynamics of dEfOM versus particulate organic matter (POM), is still lacking, and its significance has been largely overlooked.

Chin et al. provided the first evidence for the spontaneous assembly of DOM from nanogels into gels with an approximate size of 3–10 μm via cationic bridging forces and hydrophobic interactions [15]. A DOM–POM continuum is ubiquitously found in aquatic systems, including rivers, lakes, and oceans, and plays a crucial role in pollutant fate, microbial attachment, and the formation of marine snow or biofilm [6,16,17]. Larger particles (i.e., microgels) also contribute to the gravitational sedimentation of particles and scavenging of organic/inorganic pollutants [18,19,20]. For example, our previous observations demonstrated that marine and riverine microgels sequester dissolved organic matter and micro-/nano plastics, thereby altering the ultimate fate of these materials [21,22,23]. These plastics induce the DOM–POM shunt via hydrophobic interaction [24,25]. Several studies have also indicated that the aggregation and dispersion of microgels are sensitive to the surrounding environmental conditions [26,27,28]. The different sources and physicochemical characteristics of DOM are likely to influence microgel behavior and abundance [3,29,30]. The influence of temperature on marine gel dispersion most likely reflects the decreased levels of bound cationic ions in DOM, as observed by Chen et al. [31]. At present, wastewater discharge is recognized as an important driver of complex mixtures of organic matter into rivers and has unique characteristics that include severe changes (including in size, assembly speed, and structure) due to fluctuations in water conditions, especially temperature. For example, temperatures recorded in Merced, CA, USA, showed a temperature change for effluent water from 4 °C to 37 °C from winter to summer. However, most studies have focused on marine and riverine DOM systems; therefore, the current knowledge of the potential effects of dEfOM is limited. Investigation of the impacts of environmental conditions on microgel formation in effluent water is therefore needed to explore the assembly and granularity of dEfOM at different temperatures to establish the transport and fate of dEfOM in freshwater systems.

The formation behaviors in terms of the assembly of wastewater organic materials into microgels are currently unclear. The hypothesis of this study was that higher temperatures can facilitate microgel formation via hydrophobic interactions that could potentially lead to gel structure changes. In this study, we collected dEfOM samples from the wastewater outfall of the City of Merced WWTP (Merced, CA, USA) and further adjusted temperatures to examine the effects of temperature on the self-assembly kinetics, equilibrium size, and granularity of effluent microgels. The mechanism of dEfOM assembly into microgels was also investigated. The results provide information on the transformation of dEfOM (aggregation and granularity of microgels) and its transport and fate in freshwater systems.

## 2. Materials and Methods

### 2.1. Effluent Water Sampling and Water Parameter Analysis

The City of Merced WWTP (Merced, CA, USA) facility includes physical (grit classifier and clarifier etc.) processes and biological aeration basins. The effluent sample was collected at the wastewater outfall of WWTP in February 2022. After collection, the water samples were transferred to precleaned glass bottles, parafilm sealed, and stored in the dark at 4 °C until further processing. The analysis of water parameters, such as the DOC content and Ca^2+^ and Mg^2+^ concentration, was based on our previous studies [3,23]. Briefly, the effluent sample used for the determination of the dissolved organic carbon (DOC) concentration was filtered through a 0.45 μm PVDF pre-washed filter (Millipore polyvinylidene fluoride, Cork, Ireland), and DOC was measured as the total carbon by catalytic high-temperature oxidation using a Total Organic Carbon Analyzer (TOC-Vcsh, Shimadzu, Japan). The glass bottles for DOC analysis were pre-washed using 0.1 N HCl and pre-combusted at 550 °C for 4 h to avoid the contamination of the bottle by organic materials. The amount of organic carbon and cationic ions, especially Ca^2+^ and Mg^2+^, which play key roles in microgel assembly has been well established in previous studies [15]. Therefore, the background concentration of collected water needs to be provided. The amount of Ca^2+^ and Mg^2+^ was monitored by ion chromatography coupled with an IonPac CS-12A column (Dionex ICS-2000, Sunnyvale, CA, USA). The concentrations of DOC, Ca^2+^, and Mg^2+^ in the effluent water were 5.12 mg/L, 27.84 ± 0.72 mg/L, and 8.38 ± 0.06 mg/L, respectively.

### 2.2. dEfOM Sample Pretreatment and Temperature Adjustments

The effluent sample was filtered through a 0.45 μm PVDF membrane (pre-washed with 0.1 N HCl) into round-bottom polystyrene test tubes (Fisher Scientific, Waltham, MA, USA) and incubated in the dark at 4, 20, or 35 °C for 12 days, based on the wide range of temperatures recorded in Merced. Microbial activity was inhibited by adding 3 mM sodium azide (NaN_3_) to the dEfOM samples. The preparation of samples for DOM assembly in this study followed our previously described protocols [26,27,28].

### 2.3. Microgel Size and Granularity Measurements

The gel size and granularity of dEfOM samples (2 mL) were measured by flow cytometry (LSR2, BD Biosciences, Franklin Lakes, NJ, USA). The instrument was set to collect particle events for 10 min and to record signals from forward scattering and side scattering with the 488 nm laser. For quality control measurements, we used diH_2_O as blank and 0.5 μm fluorescent beads (Polysciences, Warrington, PA, USA) as controls and standards in flow cytometry checks. After particle recording, the data were analyzed with the FlowJo analysis software package (TreeStar, Woodburn, OR, USA), according to our previous research [15,28]. We collected triplicate water samples at different time points (0, 1, 3, 7, and 12 days) at the three temperature conditions and monitored the assembly rate, size distribution, and granularity of effluent microgels by flow cytometry. The *X*-axis and *Y*-axis in the quadrant are the forward and side scattering, which can represent the size and granularity of the particles.

### 2.4. Confocal Microscopy

The dEfOM samples on day 12 were stained with 30 μM Nile red and syringe-filtered through a 0.1 μm isopore membrane (Millipore, Burlington, VT, USA) [25]. The Nile red was excited with blue light (wavelength: 550 nm), and the fluorescence emission was collected at 636 nm. The images were captured with a DMi8 inverted microscope (Leica microsystems, Wetzlar, Germany). The reconstruction of the aggregate volume was based on the volume of the serial section stack.

## 3. Results and Discussion

### 3.1. Self-Assembly Kinetics and Equilibrium Size

**Figure 1:**
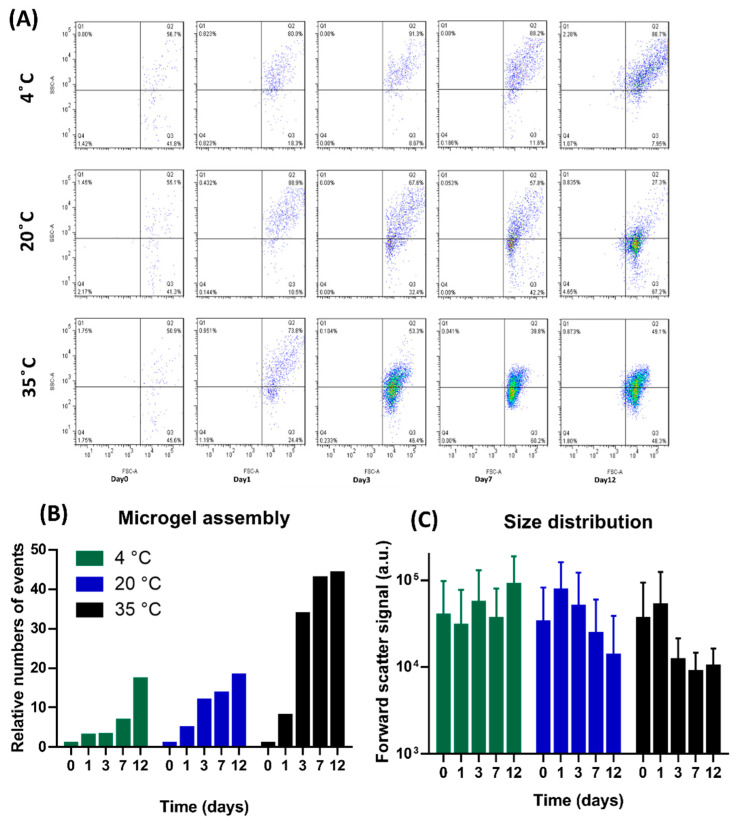
Effects of temperature on microgel assembly and size. (**A**) The microgel particle distributions in the effluent at different temperatures (4 °C, 20 °C, and 35 °C) were measured by flow cytometry. The *X*-axis represents forward scatter signal, and the *Y*-axis represents side scatter signal. The Q1 to Q4 grouping was determined by a gating strategy, and the associated numbers represent the relative population percentage. The line on the *X*-axis is defined as >0.5 μm of the particle boundary. (**B**) The relative microgel assembly numbers were calculated from the flow data. Each relative number was divided by the number of events at different temperatures on day 0. (**C**) The microgel size distribution was determined from the forward scatter signal. Each column represents mean ± SEM of forward scatter signals in the flow data.

The broad seasonal temperature differences in Merced, from 4 °C to 37 °C, prompted this investigation of a wide range of temperature effects (i.e., 4, 20, and 35 °C) on dEfOM assembly. We collected triplicate effluent samples at different time points (0, 1, 3, 7, and 12 days) at the three temperature conditions and monitored the particle abundance and distribution by flow cytometry (Figure 1A). The *X*-axis and *Y*-axis in the quadrant are the forward and side scattering, which can represent the size and granularity of the particles. Our flow cytometry data showed that particle events in the effluent water increased over time under all experimental conditions, and most of the events above the line on the *X*-axis were defined as representing >0.5 μm microgels, indicating that the dEfOM assembly formed microgels. These data suggest that dEfOM polymers can spontaneously assemble into POM, thereby reconfirming the ubiquity of the assembly of dEfOM into microgel structures in aquatic systems, including wastewater. These results are also consistent with our previous riverine observations in a polluted river [3]. The assembled microgels were found in all experiments; however, the distribution of particle events showed a distinctive pattern in the quadrant at the three temperature conditions. We further investigated the effects of temperature on the numbers and sizes of the microgels using forward scatter profiles (Figure 1B,C). When compared to day 0, the relative amounts of microgels on day 1 differed for the three temperatures. The microgels at 35 °C showed an eightfold increase, whereas the microgels at 20 °C and 4°C showed fivefold and threefold increases, respectively. The microgels formed at 35 °C underwent massive growth on day 3 (35 times) and a 43-fold increase on day 7, but the formation slowed down on day 12 (44 times). By contrast, microgels formed at 20 °C showed a steadier increase (4-, 12-, 14-, and 18-fold), and those formed at 4 °C showed relatively slower growth until day 7 (3-, 3-, and 7-fold) but a notable increase on day 12 (17-fold) (Figure 1B).

The sum of forward scatter signals is shown in Figure 1C to display the size distribution of the microgel particles. The microgels formed at the three temperatures had similar size distributions in the beginning (39,800, 33,400, and 36,500, at 4, 20, and 35 °C, respectively). The size of the microgels at 4 °C had increased gradually by day 7 (30,600, 55,600, and 36,400), but the increase was not significant until day 12 (90,000). The dimensions of the microgels at 20 °C and 35 °C rose on day 1 (77,100 and 52,100, respectively), followed by a substantial decline. The microgels at 20 °C showed a gradual size decrease with time (50,400, 24,400, and 13,800), whereas at the highest temperature of 35 °C, the microgel dimensions were strikingly reduced by day 3 (12,200) and showed a more stable and less scattered size distribution (8920 and 10,300) after that time point.

Figure 1B,C show that the extent of the assembly speed and number of microgels with time had the following order: 35 °C > 20 °C > 4 °C. By contrast, the size distribution of the formed microgels at the various temperatures showed an opposite trend: 4 °C > 20 °C > 35 °C. These observations, together with results from the number and size measurements, indicate a clear phenomenon whereby higher temperatures accelerate the self-assembly of dEfOM, but the sizes of the microgels can be reduced. These observations are similar to those reported in higher temperature environments. For example, a marine gel investigation in effluent waters near a nuclear power plant reported a positive correlation between temperature and the abundance of microgels but a negative correlation with particle size [32]. Our previous data also suggested a reduction in marine gel dimensions under simulated global warming seawaters (20–40 °C) and indicated that heated marine gels exhibited high hydrophobicity [31]. These studies suggested a possible mechanism for the findings of the present study, as higher temperatures may induce gel conformational changes that increase the hydrophobic contact area and further promote the probability of interchain bonding [33,34].

### 3.2. Granularity

**Figure 2:**
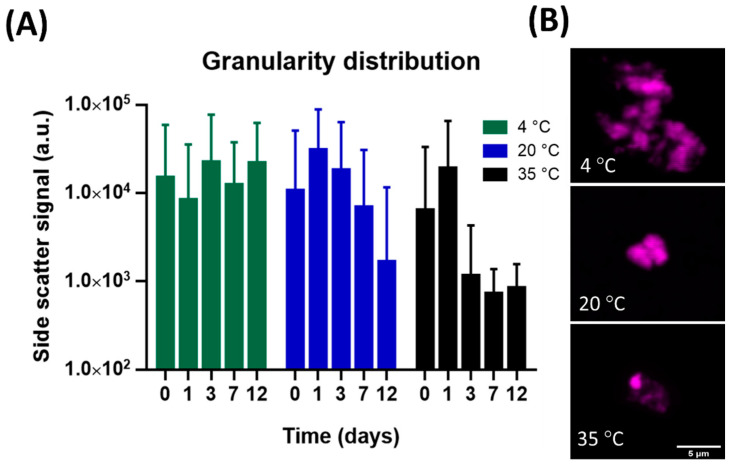
Temperature reduces microgel granularity. (**A**) The microgel granularity distribution from the flow data. Each column represents the mean ± SEM of side scatter signal in the flow data. (**B**) Confocal laser scanning micrograph showing the microgel structure and the hydrophobic sites at 4, 20, and 35 °C. Scale bar: 5 μm.

The structure of organic particles is expected to play a determining role in the sedimentation of organic particles [5,18]. Gel structure features, such as granularity, can be illustrated by the side scattering data presented in the bar graph in Figure 2A. The granularity distribution showed a similar trend to that of the microgel size distribution. Microgels at 4 °C showed equal granularity with high dispersion in the study period (15,000, 8399, 22,500, 12,400, and 22,000, at 0, 1, 3, 7, and 12 days, respectively). Microgels at 20 °C showed the highest granularity on day 1 (30,900) and declined after that time, with gradually clustered distribution (18,300, 6930, and 1660 at days 3, 7, and 12, respectively). By contrast, at 35 °C, the microgel granularity was reduced earlier on and showed a more clustered distribution. The microgel granularity at 35 °C on days 3, 7, and 12 were 1160, 727, and 842, respectively, which was the lowest side scattering observed. Nile red fluorescence staining was useful in the microgel structure observations [3,24,25]. The confocal laser scanning graphs shown in Figure 2B confirmed our conjecture and provided visual evidence for differences in the general appearance of the imaged granularity of the gels formed at 4 °C, 20 °C, and 35 °C. The gel architecture was larger but looser for the microgels formed at 4 °C than those formed at 20 °C or 35 °C.

### 3.3. Possible Mechanisms

The hypothesis of this study was that temperature increase can induce microgel formation via hydrophobic interactions that could potentially lead to gel structure changes including granularity and size distribution. Our previous data also suggested a reduction in marine gel dimensions under simulated global warming seawaters (20–40 °C) and indicated that heated marine gels exhibited high hydrophobicity [31]. These studies suggested a possible mechanism for the findings of the present study, as higher temperatures may induce gel conformational changes that increase the hydrophobic contact area and further promote the probability of interchain bonding [33,34]. Thermal-induced increases in hydrophobic contact, in combination with internal gaps in the aggregate, may be a mechanism that explains the changes in granularity alteration [35]. A temperature increase might destabilize the equilibrium hydrogen bonding of the aggregate and move hydrophilic groups, thereby allowing interactions with water. At the same time, the internal hydrophobic entanglement, which dominates the formation of compacted aggregates, would be stabilized [36]. This stable structure could rely on the hydrophilic groups to prevent the microgel from annealing to form a larger size, thereby retaining the reduced final equilibrium gel dimension at higher temperatures [15,31]. Additionally, the effluent is a complex molecular soup with multiple potential chemical and physical interactions that could influence dEfOM assembly. The role of temperature in microgel aggregation in effluents and the consequent potential for environmental effects should be addressed in future studies aimed at exploring the relationship between microgel formation and other factors, such as ions, pH, plastic interactions, and microbial effects. Knowledge of the physical characteristics of microgels, such as their density and hydrophobic/hydrophilic ratio, is also important for understanding the mechanisms of dEfOM assembly.

### 3.4. Environmental Implications

The transformation of DOM to microgels in changing environmental situations, including pollutant inputs, climate change, and ocean acidification, deserves more attention. This urgency is because gravitational sinking and transport behavior are greatly influenced by particle sizes and granularities, which also control the downward flux of organic matrices, thereby potentially disturbing carbon sequestration and biological pumps [18,37,38,39]. In addition, the aggregation of organic gels can serve as cloud condensation nuclei, so they may influence sea spray particle formation [40]. Our results revealed that higher temperatures can facilitate the assembly of dEfOM into microgel structures, but the size and granularity distributions of the microgels may decrease, suggesting that the DOM–POM shunt is sensitive to external environmental parameters. The reduction in gel sizes and the increase in their granularity could increase the buoyancy and hence the retention time of gels in the water column, potentially decreasing the sedimentation of organic particles. In a recent study, a strong enrichment of heat-reduced gel particles was observed in sea surface microlayers at high water temperatures [32,41]. The results of the present study clearly demonstrate the effects of temperature on dEfOM assembly, extending the implications to current global warming scenarios. Future studies may need to address the complex interactions occurring between the physicochemical characteristics of DOM, the different structures and sizes of microgels, and other environmental parameters that can potentially impact the dynamics of organic carbon in aquatic environments.

## 4. Conclusions

Microgel formation is an important transformation of aquatic organic matter that regulates its characteristics and structure, thereby potentially altering the fate and transport of organic matter. Our results suggest that temperature changes affected dEfOM self-assembly. A temperature of 4 °C reduces the relative assembly rate but increases the microgel size, accompanied by enhanced granularity. The size distribution of the formed microgels at the various temperatures was ordered as follows: 4 °C > 20 °C > 35 °C. By contrast, warming increases the assembly rate but produces smaller microgels with a clustered size distribution. This phenomenon has been investigated in marine gel systems and is considered to be governed by the enhancement of hydrophobic interactions. The reduction in microgel granularity by heat in the effluent sample in the present study implies that the size decrease may be caused by thermal-induced condensation.

## Data Availability

Not applicable.
